# The Association Between the Frequency of Annual Health Checks Participation and the Control of Cardiovascular Risk Factors

**DOI:** 10.3389/fcvm.2022.860503

**Published:** 2022-05-10

**Authors:** Li Lei, Yongzhen Tang, Qiuxia Zhang, Min Xiao, Lei Dai, Junyan Lu, Xinxin Lin, Xiangqi Lu, Wei Luo, Jiazhi Pan, Xiaoyu Xin, Shifeng Qiu, Yun Li, Shengli An, Jiancheng Xiu

**Affiliations:** ^1^Department of Cardiology, Nanfang Hospital, Southern Medical University, Guangzhou, China; ^2^Department of Cardiology, Nanfang Hospital Zengcheng Branch, Guangzhou, China; ^3^Department of Public Health Management, Zengcheng Xintang Hospital, Guangzhou, China; ^4^Department of Biostatistics, School of Public Health, Southern Medical University, Guangzhou, China

**Keywords:** basic public health service, annual health checks, cardiovascular disease, risk factors, cross-sectional study

## Abstract

**Background:**

General health checks can help in controlling cardiovascular risk factors. However, few studies have investigated whether regular participation in annual health checks could further improve the control of cardiovascular risk factors compared with intermittent participation. Therefore, our study aimed to explore the association between the frequency of annual health check participation and the control of cardiovascular risk factors.

**Methods:**

Residents aged ≥ 65 years or having chronic diseases (hypertension or diabetes) from 37 communities of Guangzhou, Guangdong, who participated in the Basic Public Health Service project between January 2015 and December 2019, were enrolled and divided into 3 groups (“Sometimes,” “Usually,” and “Always”) according to their frequencies of annual health check participation. Multivariable linear regression models were performed to assess the association between the frequency of annual health check participation and the control of cardiovascular risk factors. A subgroup analysis stratified by gender was also conducted.

**Results:**

In total, 9,102 participants were finally included. Significant differences were identified between groups in systolic blood pressure (SBP), diastolic blood pressure (DBP), weight, fasting glucose, total cholesterol, high-density lipoprotein cholesterol, and serum creatinine. After fully adjusting for confounding factors, residents who always participated in the annual health check tended to have lower SBP (β = −4.36, 95% *CI*: −5.46; −3.26, *p* < 0.001), fasting glucose (β = −0.27, 95% *CI*: −0.38; −0.15, *p* < 0.001), and total cholesterol (β = −0.19, 95% CI: −0.26; −0.13, *p* < 0.001), compared with those who attended sometimes. Furthermore, gender did not alter these associations.

**Conclusion:**

A higher frequency of annual health check participation was associated with lower SBP, fasting glucose, and total cholesterol.

## Introduction

Cardiovascular diseases (CVDs) are the leading cause of death globally, with an estimated 18.6 million CVD deaths in 2019 (1, 2). In China, there are currently about 330 million patients suffering from CVDs, including 13 million patients with stroke and 11.39 million patients with coronary heart disease, which has caused great burdens on society (3). To improve health service continuity and regional equality, the Chinese government has launched the Basic Public Health Service (BPHS) project in 2009, which provides a free annual health check for older adults (≥ 65 years old) and patients with chronic disease (hypertension and diabetes) (4). However, due to the lack of health consciousness, most residents do not attend the annual health checks regularly and on time.

Although general health checks failed to reduce mortality or cardiovascular events, they were able to improve risk factor control (5). In the United Kingdom Family Heart Study, 12,472 men and women were enrolled and randomly assigned to receive a general health check with a follow-up nurse led lifestyle intervention or usual care (no health check or intervention). After 1 year, participants who received the health check and intervention demonstrated lower systolic blood pressure (SBP) [men: 131.6 vs. 139.0 mmHg; difference (SE), −7.3 (0.8); women: 123.2 vs. 129.6 mmHg; difference (SE), −6.2 (0.9)] and diastolic blood pressure (DBP) [men: 83.3 vs. 86.6 mmHg; difference (SE), −3.5 (0.4); women: 78.6 vs. 81.3 mmHg; difference (SE), −3.0 (0.4)] compared with those who received usual care (6). Similar results were observed in the Minnesota Heart Health Project and the OXCHECK study in which a single general health check could lead to lower blood pressure and cholesterol (7, 8). Though several studies reported that general health checks can improve risk factor control, no study has investigated whether active and regular participation in annual health checks can further improve the control of cardiovascular risk factors compared with intermittent participation. Therefore, our study aimed to explore the association between the frequency of annual health checks participation and the control of cardiovascular risk factors.

## Materials and Methods

### Participants

Residents from 37 communities of Guangzhou, Guangdong, who participated in the BPHS between January 2015 and December 2019, were enrolled. To participate in the BPHS, one must meet at least one of the following inclusion criteria: (1) age ≥ 65 years; (2) having hypertension; and (3) having diabetes mellitus. Hypertension and diabetes mellitus were diagnosed by general practitioners according to the published guidelines (9, 10). This study was approved by the Ethics Committee of the Nanfang Hospital (NFEC-2021-083).

### Health Check Contents and Data Collection

The Chinese BPHS project provides a general health check for older adults and patients with chronic disease (hypertension or diabetes mellitus) annually. The health check contents include a physical examination, lifestyle questionnaires, blood tests, and an electrocardiogram (ECG). All health checks data were recorded in a regional chronic disease management platform, and data from this platform between January 2015 and December 2020 were obtained. For individuals who participated in the Chinese BPHS project more than once over this time period, data from their most recent participation were used for analyses. In addition, for the current analysis, patients with missing laboratory examination data missing in their latest health check were excluded.

### Definitions

The risk factors of CVD concerned in this study were mainly anthropometric or laboratory indexes of health check content that are modifiable, such as blood pressures, lipid profiles, weight, etc. Patients were deemed as having atrial fibrillation (AF) if they were diagnosed with atrial fibrillation through a 12-lead ECG according to the guidelines during any of the annual health checks they participated in (11). Chronic kidney disease (CKD) was defined as an estimated glomerular filtration rate (eGFR) of less than 60 ml/min/1.73 m^2^, where eGFR was calculated using the Modification of Diet in Renal Disease (MDRD) equation (12, 13).

### Statistical Analysis

All included participants were divided into 3 groups (“Sometimes,” “Usually,” and “Always”) according to their frequencies of annual health checks participation which was calculated through the following equation:

Frequency of annual health checks participation = Times of annual health check participation from the year of first annual health check participation to 2020/number of years between the year of first annual health check participation and 2020.

For example, a resident participated in the annual health check for the first time in 2017 and again in 2019 and 2020. Then, the resident’s frequency of annual health check participation was 3/4. “Always” group, defined as the frequency of annual health check participation = 1, refers to residents in this group who regularly participated in the annual health check and were never absent. “Usually” was defined as the frequency of annual health check participation > 0.5 and < 1, and the “Sometimes” group was defined as frequency ≤ 0.5.

Continuous variables were expressed as mean ± SD or median [interquartile] and were compared by using the analysis of variance (ANOVA). Categorical variables were expressed as percentages and compared using the Pearson χ^2^ test. ANOVA with the *post hoc* Bonferroni correction was conducted to evaluate the difference between the 3 groups in cardiovascular risk factors. Three multivariable linear regression models were performed to assess the association between the frequency of annual health check participation and the control of cardiovascular risk factors. The first model was adjusted for age and sex. The second model was adjusted for age, sex, smoking, drinking, and exercise, and the third model was adjusted for all the variables in model 2 plus serum creatinine, statin, hypertensive treatment, and hypoglycemic treatment. To explore the linear trends, we ran linear or logistic regression models adjusted for age and sex, in which health check frequency was entered as an ordinal variable. In addition, the gender difference was explored. The value of *p* < 0.05 was considered statistically significant. All analyses were conducted with SPSS (version 25.0) and R software (version 4.1.0).

## Results

### Characteristics Stratified by Health Check Frequency

**FIGURE 1:**
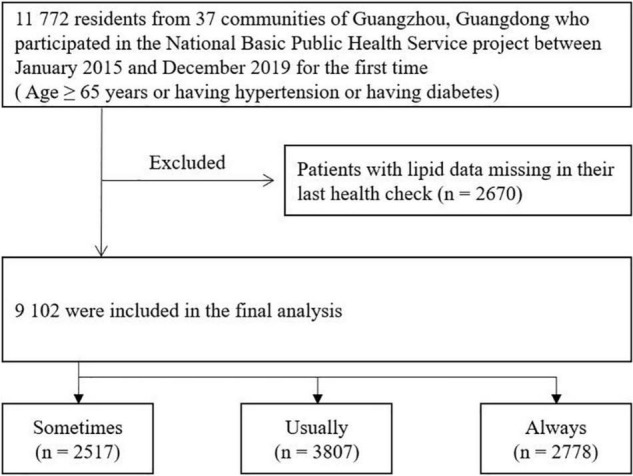
Flowchart of the case selection progress.

From January 2015 to December 2019, 11,772 residents participated in the annual health checks of the BPHS project, among which 2,670 had their laboratory examinations data missing in their latest health check between January 2015 and December 2020. Therefore, 9,102 participants were finally included in the current analysis (Figure 1).

Generally, there were 3,578 men (39.31%), with an average age of 71.65 ± 6.55 years. The characteristics of the included participants are listed in Table 1. From 2015 to 2020, the median times of annual health check participation were 2 [2; 3], 4 [4; 5], and 6 [4; 6] for the “Sometimes” group, the “Usually” group, and the “Always” group, respectively. Compared with participants in other groups, those in the “Always” group were older, had more women, hypertension, and were less likely to be smokers and drinkers. Significant differences between groups were also identified in exercise habits and hypertensive treatment.

**TABLE 1:** Characteristics stratified by health check frequency.

Variables	Missing value, n (%)	Overall (*n* = 9,102)	Sometimes (*n* = 2,517)	Usually (*n* = 3,807)	Always (*n* = 2,778)	*P* value
Age, years	0 (0.00)	71.65 ± 6.55	69.59 ± 6.96	72.16 ± 6.49	72.84 ± 5.79	<0.001
Heart rate, bpm	2 (0.02)	74.13 ± 12.34	75.66 ± 12.78	74.06 ± 12.34	72.84 ± 11.78	<0.001
Male, n (%)	0 (0.00)	3,578 (39.31)	1,027 (40.80)	1,496 (39.30)	1,055 (37.98)	0.110
Hypertension, n (%)	0 (0.00)	4,634 (52.66)	1,157 (48.57)	1,949 (51.53)	1,528 (57.97)	<0.001
Diabetes, n (%)	0 (0.00)	1,607 (18.51)	432 (18.50)	691 (18.34)	484 (18.78)	0.905
Tobacco, n (%)	2 (0.02)					<0.001
Never		7,376 (81.05)	1,946 (77.34)	3,107 (81.61)	2,323 (83.65)	
Used to smoke		690 (7.58)	240 (9.54)	278 (7.30)	172 (6.19)	
Current smoker		1,034 (11.36)	330 (13.12)	422 (11.08)	282 (10.15)	
Drinking, n (%)	1 (0.01)					<0.001
Never		7,846 (86.21)	2,114 (84.02)	3,264 (85.74)	2,468 (88.84)	
Sometimes		813 (8.93)	256 (10.17)	346 (9.09)	211 (7.60)	
Often		74 (0.81)	22 (0.87)	39 (1.02)	13 (0.47)	
Everyday		368 (4.04)	124 (4.93)	158 (4.15)	86 (3.10)	
Exercise, n (%)	0 (0.00)					<0.001
Never		2,789 (30.64)	900 (35.76)	1,118 (29.37)	771 (27.75)	
Sometimes		872 (9.58)	213 (8.46)	387 (10.17)	272 (9.79)	
At least once a week		170 (1.87)	61 (2.42)	67 (1.76)	42 (1.51)	
Everyday		5,271 (57.91)	1,343 (53.36)	2,235 (58.71)	1,693 (60.94)	
Hypertensive treatment	154 (1.69)	4,281 (47.84)	986 (41.13)	1,830 (48.41)	1,465 (52.87)	<0.001
Hypoglycemic treatment	117 (1.29)	1,462 (16.27)	371 (15.28)	644 (17.01)	447 (16.13)	0.191
Statin	94 (1.03)	44 (0.49)	15 (0.61)	20 (0.53)	9 (0.32)	0.298
Times of health check participation	0 (0.00)	4.00 [3.00, 5.00]	2.00 [2.00, 3.00]	4.00 [4.00, 5.00]	6.00 [4.00, 6.00]	<0.001

### Cardiovascular Risk Factors Stratified by Health Check Frequency

**FIGURE 2:**
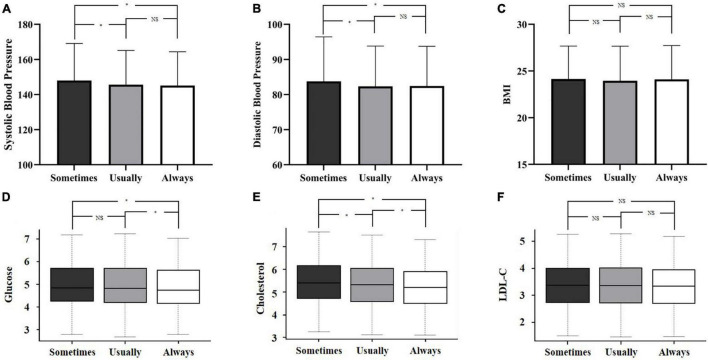
Classic cardiovascular risk factors stratified by health check frequency. Differences in systolic blood pressure **(A)**, diastolic blood pressure **(B)**, BMI **(C)**, glucose **(D)**, cholesterol **(E)**, and LDL-C **(F)** between different health check frequencies. *p < 0.05. NS, not significant; BMI, body mass index; LDL-C, low-density lipoprotein cholesterol.

As shown in Table 2, significant differences were identified between three health check frequency groups in SBP, DBP, weight, fasting glucose, total cholesterol, high-density lipoprotein cholesterol, and serum creatinine. Linear analysis revealed that higher health check frequency has linear trends with lower SBP, weight, fasting glucose, and total cholesterol but higher serum creatinine (all *p* for trend < 0.05). The pairwise comparison between groups showed a significant difference in SBP when comparing the “Usually” group or the “Always” group to the “Sometimes” group, whereas no significant difference was found between the “Usually” group and the “Always” group. The same result was also observed in DBP. As for fasting glucose, patients in the “Always” group tended to have a lower level when compared to the other two groups. Besides, for total cholesterol, remarkable differences were identified between the three groups (Figure 2).

**TABLE 2:** Cardiovascular risk factors stratified by health check frequency.

Variables	Missing value, n (%)	Sometimes (*n* = 2,517)	Usually (*n* = 3,807)	Always (*n* = 2,778)	*P* value	P for trend*
SBP, mmHg	4 (0.04)	147.99 ± 21.12	145.62 ± 19.51	145.08 ± 19.32	< 0.001	<0.001
DBP, mmHg	9 (0.10)	83.77 ± 12.66	82.33 ± 11.48	82.42 ± 11.33	< 0.001	0.185
Weight, kg	9 (0.10)	59.15 ± 10.30	58.45 ± 10.84	58.43 ± 10.24	0.017	0.006
BMI, kg/m^2^	9 (0.10)	24.14 ± 3.53	23.95 ± 3.71	24.10 ± 3.63	0.095	0.028
Waist, cm	11 (0.12)	85.70 ± 9.40	85.71 ± 9.77	85.73 ± 9.49	0.995	0.601
Fasting glucose, mmol/L	2 (0.02)	4.83 [4.25, 5.71]	4.80 [4.19, 5.70]	4.74 [4.16, 5.60]	0.001	< 0.001
Creatinine, μmol/L	0 (0.00)	75.21 [63.20, 90.92]	79.85 [66.92, 95.70]	82.03 [69.74, 96.96]	< 0.001	0.004
eGFR, mL/min/1.73 m^2^	0 (0.00)	80.25 [66.25, 93.80]	74.07 [61.43, 86.71]	70.89 [60.19, 83.22]	< 0.001	0.238
Total cholesterol, mmol/L	1 (0.01)	5.40 [4.71, 6.17]	5.31 [4.57, 6.04]	5.20 [4.50, 5.90]	< 0.001	<0.001
Triglyceride, mmol/L	2 (0.02)	1.44 [1.03, 2.13]	1.43 [1.02, 2.09]	1.46 [1.04, 2.10]	0.822	0.781
LDL-C, mmol/L	4 (0.04)	3.36 [2.74, 4.00]	3.36 [2.73, 4.00]	3.34 [2.70, 3.94]	0.254	0.291
HDL-C, mmol/L	17 (0.19)	1.31 [1.09, 1.57]	1.33 [1.11, 1.61]	1.32 [1.09, 1.59]	0.042	0.769
Uric acid, μmol/L	6,318 (69.41)	372.00 [305.86, 444.00]	376.73 [319.36, 451.16]	378.00 [317.91, 455.00]	0.536	0.577
AF, n (%)	24 (0.26)	58 (2.33)	105 (2.76)	60 (2.16)	0.267	0.122
CKD, n (%)	0 (0.00)	407 (16.17)	851 (22.35)	686 (24.69)	< 0.001	<0.001

*Adjusted for age and sex for the trend.

*SBP, systolic blood pressure; DBP, diastolic blood pressure; BMI, body mass index; eGFR, estimated glomerular filtration rate; LDL-C, low-density lipoprotein cholesterol; HDL-C, high-density lipoprotein cholesterol; AF, atrial fibrillation; and CKD, chronic kidney disease.*

The association between the health check frequency and the control of cardiovascular risk factors is displayed in Table 3. After fully adjusting for confounding factors, residents who attended annual health checks more frequently had lower SBP (β for the “Usually” group = −3.37, *p* < 0.001; β for the “Always” group = −4.36, *p* < 0.001), and lower total cholesterol (β for the “Usually” group = −0.10, *p* < 0.001; β for the “Always” group = −0.19, *p* < 0.001), compared to those who attended occasionally. Attending annual health checks usually rather than always was observed to be associated with lower DBP, whereas attending always rather than usually was associated with lower fasting glucose. No significant association was observed between the health check frequency and body mass index (BMI) and low-density lipoprotein cholesterol.

**TABLE 3:** Multivariable linear regression analysis of the association between the frequency of annual health check participation and the control of cardiovascular risk factors.

Variables	Sometimes	Usually		Always	
			
		β (95% CI)	*P* value	β (95% CI)	*P* value
Systolic blood pressure, mmHg					
Model I	Reference	−3.22 (−4.23; −2.21)	<0.001	−3.99 (−5.08; −2.90)	<0.001
Model II	Reference	−3.31 (−4.32; −2.30)	<0.001	−4.02 (−5.12; −2.94)	<0.001
Model III	Reference	−3.37 (−4.39; −2.35)	<0.001	−4.36 (−5.46; −3.26)	<0.001
Diastolic blood pressure, mmHg					
Model I	Reference	−0.75 (−1.35; −0.16)	0.013	−0.46 (−1.10; 0.18)	0.155
Model II	Reference	−0.81 (−1.41; −0.22)	0.008	−0.49 (−1.13; 0.15)	0.132
Model III	Reference	−0.81 (−1.42; −0.21)	0.008	−0.60 (−1.25; 0.05)	0.071
Body mass index, kg/m^2^					
Model I	Reference	0.02 (−0.16; 0.21)	0.809	0.22 (0.02; 0.41)	0.032
Model II	Reference	0.02 (−0.17; 0.20)	0.849	0.21 (0.01; 0.41)	0.038
Model III	Reference	−0.04 (−0.22; 0.14)	0.683	0.10 (−0.10; 0.30)	0.317
Fasting glucose, mmol/L					
Model I	Reference	−0.09 (−0.20; 0.02)	0.112	−0.26 (−0.38; −0.14)	<0.001
Model II	Reference	−0.09 (−0.21; 0.02)	0.106	−0.26 (−0.38; −0.13)	<0.001
Model III	Reference	−0.13 (−0.23; −0.02)	0.015	−0.27 (−0.38; −0.15)	<0.001
Total cholesterol, mmol/L					
Model I	Reference	−0.11 (−0.16; −0.05)	<0.001	-0.21 (−0.27; −0.15)	<0.001
Model II	Reference	−0.11 (−0.17; −0.06)	<0.001	−0.21 (−0.28; −0.15)	<0.001
Model III	Reference	−0.10 (−0.16; −0.04)	<0.001	−0.19 (−0.26; −0.13)	<0.001
Low-density lipoprotein cholesterol, mmol/L					
Model I	Reference	0.00 (−0.05; 0.05)	0.974	−0.03 (−0.08; 0.02)	0.305
Model II	Reference	−0.01 (−0.05; 0.04)	0.800	−0.03 (−0.09; 0.02)	0.199
Model III	Reference	0.00 (−0.05; 0.04)	0.856	−0.03 (−0.08; 0.02)	0.277

*Model I: adjusted for age and sex;*

*Model II: adjusted for age and sex, smoking, drinking, and exercise;*

*Model III: adjusted for age, sex, smoking, drinking, exercise, serum creatinine, statin, hypertensive treatment, and hypoglycemic treatment.*

### Stratified Analysis by Gender

Since 60.69% of the enrolled participants were women, we conducted further analysis stratified by gender to explore whether the association between the frequency of annual health checks participation and the control of cardiovascular risk factors differs between men and women.

Regardless of gender, residents who always participated in the annual health checks tended to have lower SBP, fasting glucose, and total cholesterol compared to those who attended sometimes after adjusting for confounding factors (Supplementary Table 1). Moreover, male participants in the “Always” group seemed to have a greater reduction in SBP than female participants in the “Always” group (*p* for interaction = 0.031).

## Discussion

Based on 9,102 residents from 37 communities of Guangzhou, Guangdong, who participated in the BPHS between January 2015 and December 2019, our study first explored the association between the frequency of annual health check participation and the control of cardiovascular risk factors in older adults and patients with hypertension or diabetes. Results showed that more frequent participation in the annual health checks was associated with lower SBP, fasting glucose, and total cholesterol regardless of gender, whereas no significant association was observed between the health check frequency and the BMI and low-density lipoprotein cholesterol.

In 2009, the Chinese government launched the BPHS project which provides a free health check for older adults and patients with chronic diseases (hypertension and diabetes) annually (4). However, except for those who have never participated in the annual health check, even residents who have ever attended, only 30.52% of them attended regularly without being absent. Previous studies showed that general health checks can improve risk factor control (5). Furthermore, in our study, we found that, regardless of gender, residents who participated in the annual health checks regularly without being absent tended to have lower SBP, fasting glucose, and total cholesterol than those who attended sometimes. These findings were an extension of the previous results (7, 8, 14). Murray et al. enrolled 2,323 community residents in the United States and randomly (1:1) assigned them to intervention group (risk factor screening and health education) or control group (nothing). After 1 year of intervention, participants in the intervention group had lower total cholesterol, DBP, and heart rate (7). A retrospective observational study with a median follow-up time of 2 years enrolled 138,788 patients aged 40–74 years and without a previous diagnosis of vascular disease. After matching, attendees of the National Health Service Health Check program had a significant absolute reduction in SBP (−2.51 mmHg, 95% *CI* −2.77 to −2.25 mmHg), DBP (−1.46 mmHg, 95% *CI* −1.62 to −1.29 mmHg), BMI (−0.27, 95% *CI* −0.34 to −0.20), and total cholesterol (−0.15 mmol/L, 95% *CI* −−0.18 to −0.13 mmol/L) (15). In contrast to these previous studies, the control of the current study involved residents who intermittently rather than never participated in the annual health checks. Therefore, together with the previous results, our findings indicated that annual health checks can improve risk factor control and taking annual health checks regularly may even further strengthen this improvement.

Previous studies have proven that SBP is positively associated with cardiovascular events (16–18). A recent study involving 157,728 participants with previous CVDs and 186,988 participants without previous CVDs found that a reduction of SBP by 5 mmHg is associated with 9% fewer major cardiovascular events [hazard ratio (*HR*): 0.91, 95% *CI*: 0.89–0.94] for participants without previous CVDs and 11% fewer major cardiovascular events (*HR*: 0.89, 95% *CI*: 0.86–0.92) for those with previous CVDs (16). In addition, a meta-analysis revealed that SBP reduction by 5 mmHg reduced the risk of incident type 2 diabetes by 11% (*HR*: 0.89, 95% *CI*: 0.86–0.92) (19). In our study, after adjusting for confounding factors, residents who always participated in the annual health checks tended to have lower SBP (β:−4.36, 95% *CI*: −5.46 ∼−3.26) than those who attended sometimes. Though the degree of decline in SBP is not so much as that of medication treatment, it may help in reducing clinical events.

Moreover, it is worth mentioning that some earlier randomized controlled studies, which were conducted before 1990 when statins were not popular, confirmed that general health checks can improve cholesterol control (7, 20). In our study, we also found that a higher frequency of annual health check participation was associated with lower total cholesterol, which was independent of statin treatment. Since regularly participating in annual health checks often means better health consciousness, we considered that the potential effect of general health checks on cholesterol control is mainly based on better health consciousness and health behaviors rather than statin treatment (8, 21). However, new antilipemic drugs, such as proprotein convertase subtilisin-kexin type 9 (PCSK9) inhibitors, are becoming more and more readily available. Though they are currently not widely prescribed in primary care, they may modify the impact of health check frequency on lipid control in the near future (22, 23). In addition, we noticed that this significant impact exists only in total cholesterol but not in low-density lipoprotein cholesterol or triglyceride. The potential reasons may be that the BPHS project in China provides health checks and result feedback with no systematic health education for participants. Therefore, the intervention of the project on the lifestyles and health awareness of the participants is limited. It cannot lead to tremendous differences in lipid profiles. Compared with low-density lipoprotein cholesterol, total cholesterol refers to all cholesterol contained in lipoproteins in the blood, which means it is easier to be influenced. Furthermore, though the frequency of health check participation was not significantly associated with lower low-density lipoprotein cholesterol or triglycerides in the three adjusted linear regression models, it demonstrated a potentially negative correlation. The statistical insignificance may be partly explained by the insufficient sample size.

As for atrial fibrillation detection, previous studies demonstrated that general health checks were associated with the increased detection of chronic disease (diabetes, hypertension, and CKD) (5). Moreover, longer screening times may increase the detection rate of AF (24). However, in this study, no significant difference was observed in the detection rate of AF between the 3 groups. Additionally, no significant association was observed between the health check frequency and AF detection in the multivariable logistic regression. The reason for this phenomenon may be that, during each annual health check, participants only received a 12-lead ECG screening for up to 15 s. That means the difference in the screening time between groups was only about 30 s. Such difference may be too little to cause differences in the detecting rate of AF between groups.

In this study, we also found that residents who participated in the annual health checks more frequently tended to have a higher prevalence of CKD. As was mentioned above, the BPHS project in China only provides health checks and result feedback. There is a lack of systematic management. Recently, the PONTE-SCA Puglia program introduced integrated management for patients with acute coronary syndrome to strengthen their adherence to recommended therapies, and reduce the incidence of adverse events. This integrated management involving both general hospitals and primary medical institutions is worthy of reference in the future development of the BPHS project in China (25). Moreover, with the engagement of professional care managers in the future, the need for concentrative annual health checks may also be reduced.

This study also has several limitations. First, the enrolled population was based on 37 communities of Guangzhou, Guangdong, and there is a risk of lacking representativeness. Second, due to the cross-sectional design and limited data, we were not able to explore the association between the frequency of the health checks and several other important risk factors, for example, incident diabetes for non-diabetics and HbA1c. Third, the current results should be interpreted with caution due to the cross-sectional design. Since we were not able to explore the causal association between the frequency of annual health check participation and the control of cardiovascular risk factors, further prospective longitudinal studies are needed to confirm these observations.

## Conclusion

A higher frequency of annual health check participation was associated with lower SBP, fasting glucose, and total cholesterol regardless of gender. Together with the previous studies, our findings indicated that taking annual health checks regularly may even further improve the control of cardiovascular risk factors. However, further prospective longitudinal studies are needed to confirm these observations.

## Data Availability Statement

Data relevant to this study are available upon reasonable request to the corresponding authors.

## Ethics Statement

The studies involving human participants were reviewed and approved by the Ethics Committee of the Nanfang Hospital. The patients/participants provided their written informed consent to participate in this study.

## Author Contributions

LL, QZ, and JX conceived and designed the study. LL, QZ, YT, MX, LD, JL, XXL, XQL, JP, XX, SQ, YL, and WL performed the acquisition and analysis of data. LL, QZ, and SA contributed to statistical analysis of data and interpretation. LL, YT, and QZ wrote the manuscript. SA and JX revised the manuscript. All authors read and approved the final version of the manuscript.

## Conflict of Interest

The authors declare that the research was conducted in the absence of any commercial or financial relationships that could be construed as a potential conflict of interest.

## Publisher’s Note

All claims expressed in this article are solely those of the authors and do not necessarily represent those of their affiliated organizations, or those of the publisher, the editors and the reviewers. Any product that may be evaluated in this article, or claim that may be made by its manufacturer, is not guaranteed or endorsed by the publisher.
