# Supplementary material for: The Association Between the Frequency of Annual Health Checks Participation and the Control of Cardiovascular Risk Factors
Source: Front Cardiovasc Med. 2022 May 10;9:860503. doi: 10.3389/fcvm.2022.860503 (PMC9127134; doi:10.3389/fcvm.2022.860503)
Supplement: Supplementary file 1 [file Table_1.DOCX]

Supplementary Material

**Supplementary Table 1. Gender stratified analysis of the association between the frequency of annual health checks participation and the control of cardiovascular risk factors.**

| Variables | Sometimes | Usually | | Always | | P for interaction |
| --- | --- | --- | --- | --- | --- | --- |
|  |  | β (95% CI) | P value | β (95% CI) | P value |  |
| Systolic blood pressure | | | | | | 0.031 |
| Male | Reference | -4.56 (-6.17; -2.96) | < 0.001 | -5.03 (-6.78; -3.29) | < 0.001 |  |
| Female | Reference | -2.54 (-3.86; -1.22) | < 0.001 | -3.88 (-5.29; -2.46) | < 0.001 |  |
| Diastolic blood pressure | | | | | | 0.372 |
| Male | Reference | -1.13 (-2.10; -0.15) | 0.023 | -0.89 (-1.95; 0.17) | 0.100 |  |
| Female | Reference | -0.59 (-1.37; 0.18) | 0.132 | -0.39 (-1.22; 0.44) | 0.354 |  |
| Body mass index | | | | | | 0.055 |
| Male | Reference | 0.18 (-0.08; 0.45) | 0.182 | 0.11 (-0.18; 0.40) | 0.468 |  |
| Female | Reference | -0.18 (-0.42; 0.07) | 0.155 | 0.09 (-0.17; 0.36) | 0.495 |  |
| Fasting glucose | | | | | | 0.378 |
| Male | Reference | -0.19 (-0.36; -0.02) | 0.027 | -0.28 (-0.47; -0.10) | 0.002 |  |
| Female | Reference | -0.09 (-0.21; 0.04) | 0.189 | -0.26 (-0.40; -0.12) | < 0.001 |  |
| Total cholesterol | | | | | | 0.151 |
| Male | Reference | -0.15 (-0.24; -0.06) | < 0.001 | -0.20 (-0.30; -0.10) | < 0.001 |  |
| Female | Reference | -0.06 (-0.14; 0.01) | 0.091 | -0.19 (-0.27; -0.11) | < 0.001 |  |
| Low-density lipoprotein cholesterol | | | | | | 0.066 |
| Male | Reference | -0.07 (-0.14; 0.01) | 0.084 | -0.04 (-0.12; 0.04) | 0.296 |  |
| Female | Reference | 0.04 (-0.03; 0.10) | 0.267 | -0.02 (-0.09; 0.05) | 0.588 |  |

Adjusted for age, smoking, drinking, exercise, serum creatinine, statin, hypertensive treatment and hypoglycemic treatment.
